# Targeting the Intrinsic Apoptosis Pathway: A Window of Opportunity for Prostate Cancer

**DOI:** 10.3390/cancers14010051

**Published:** 2021-12-23

**Authors:** Daniel Westaby, Juan M. Jimenez-Vacas, Ana Padilha, Andreas Varkaris, Steven P. Balk, Johann S. de Bono, Adam Sharp

**Affiliations:** 1 Division of Clinical Studies, The Institute of Cancer Research, London SM2 5NG, UK; daniel.westaby@icr.ac.uk (D.W.); juan.jimenezvacas@icr.ac.uk (J.M.J.-V.); ana.primpadilha@icr.ac.uk (A.P.) Johann.DeBono@icr.ac.uk (J.S.d.B.); 2 Prostate Cancer Targeted Therapy Group, The Royal Marsden Hospital, London SM2 5PT, UK; 3 Hematology-Oncology Division, Beth Israel Deaconess Medical Center, Boston, MA 02215, USA; avarkaris@mgh.harvard.edu (A.V.); sbalk@bidmc.harvard.edu (S.P.B.)

**Keywords:** prostate cancer, cell death, apoptosis, BH3 mimetics

## Abstract

**Simple Summary:**

Prostate cancer treatment has improved over the last 20 years; despite this, approximately 33,000 men died from the disease in the United States in 2020. In view of this, new treatment options are urgently needed for advanced prostate cancer. Eradicating cancer cells by triggering apoptosis (a form of cell death) is an attractive strategy, and a novel class of drugs, called BH3 mimetics, have been designed to do this. They have been shown to work for blood cancers and may also have a role in solid cancers. Herein, we discuss cell death, focusing on the intrinsic apoptosis pathway, and consider how BH3 mimetics may be used to help treat prostate cancer.

**Abstract:**

Despite major improvements in the management of advanced prostate cancer over the last 20 years, the disease remains invariably fatal, and new effective therapies are required. The development of novel hormonal agents and taxane chemotherapy has improved outcomes, although primary and acquired resistance remains problematic. Inducing cancer cell death via apoptosis has long been an attractive goal in the treatment of cancer. Apoptosis, a form of regulated cell death, is a highly controlled process, split into two main pathways (intrinsic and extrinsic), and is stimulated by a multitude of factors, including cellular and genotoxic stress. Numerous therapeutic strategies targeting the intrinsic apoptosis pathway are in clinical development, and BH3 mimetics have shown promising efficacy for hematological malignancies. Utilizing these agents for solid malignancies has proved more challenging, though efforts are ongoing. Molecular characterization and the development of predictive biomarkers is likely to be critical for patient selection, by identifying tumors with a vulnerability in the intrinsic apoptosis pathway. This review provides an up-to-date overview of cell death and apoptosis, specifically focusing on the intrinsic pathway. It summarizes the latest approaches for targeting the intrinsic apoptosis pathway with BH3 mimetics and discusses how these strategies may be leveraged to treat prostate cancer.

## 1. Introduction

Prostate cancer is the most frequently occurring cancer in men in the Western world, and was responsible for approximately 330,000 deaths in the United States in 2020 [1,2]. Around three-quarters of men with prostate cancer are diagnosed with localized disease and can be treated with curative intent; most commonly using a radical prostatectomy or radiotherapy [3]. However, an increasing number, and percentage, of men are being diagnosed with de novo metastatic disease, and relapse occurs in 20 to 50% after radical treatment of early-stage disease [3,4,5,6,7]. Prostate cancer is predominantly an androgen-dependent disease, and androgen deprivation therapy (ADT; herein defined as medical or surgical castration) has remained the backbone of treatment for advanced disease since 1941, when Charles Huggins and Clarence Hodges discovered its beneficial effect for men with metastatic disease [8,9,10,11]. 

The majority of advanced prostate cancers respond to ADT, but they inevitably progress from castration-sensitive prostate cancer (CSPC) to castration-resistant prostate cancer (CRPC), which is driven by a range of androgen receptor (AR)-dependent and independent mechanisms (e.g., *AR* amplification and neuroendocrine trans-differentiation, respectively) [12]. Despite significant progress in management over the last 20 years, advanced CRPC remains lethal, with a poor prognosis and an estimated median survival of between 2 and 3 years [13,14]. Life-prolonging treatments for advanced CRPC include taxane-based chemotherapy (docetaxel and cabazitaxel), novel hormonal agents (abiraterone and enzalutamide), a bone specific alpha-emitting radionuclide (radium-223), and an autologous cellular immunotherapy (sipuleucel-T) [15,16,17,18,19,20,21,22,23]. These agents were initially approved for advanced CRPC, though subsequent clinical trials have shown that docetaxel and novel hormonal agents (abiraterone, enzalutamide, and apalutamide) are also effective when utilized earlier in the disease course for advanced CSPC [24,25,26,27,28,29].

Advanced prostate cancer is a heterogenous disease with a multitude of primary and acquired treatment resistance mechanisms [30,31]. Molecular characterization and the identification of clinically useful predictive biomarkers is of high importance for patient selection. In this space, in recent years, aberrations in homologous recombination repair (HRR) and DNA damage response (DDR) genes have been shown to sensitize tumors to poly(ADP-ribose) polymerase (PARP) inhibitors via synthetic lethality, and have subsequently been approved by the FDA for this subset of metastatic CRPC [32,33]. PTEN loss is emerging as a predictive biomarker for response to AKT inhibition, and the anti-PD-1 agent pembrolizumab has been approved for all advanced solid tumors with defective mismatch repair or microsatellite instability-high, including advanced prostate cancer [34,35,36]. PSMA (prostate-specific membrane-antigen) is highly expressed in most prostate cancers, especially in CRPC [37,38]. ^177^Lutetium-PSMA-617, a PSMA ligand labelled with a beta-emitting radionuclide lutetium-177, accumulates at PSMA expressing tumor sites, inducing cancer cell death, and has recently been shown to be effective for men with advanced PSMA-positive CRPC [39]. Although these therapies have improved outcomes for men with advanced prostate cancer, none of them are curative, and new effective treatment strategies are urgently required.

Apoptosis, a form of regulated cell death, can be divided into the intrinsic and extrinsic pathways. Established therapies for advanced prostate cancer, including ADT and chemotherapy, induce cellular stress and can subsequently activate the intrinsic apoptosis pathway, but have limited success in doing this [40,41,42]. Although ADT does induce apoptosis in a minority of prostate cancers, the predominant mechanisms of tumor regression are reduced proliferation, cell cycle arrest, and senescence, allowing cancer cells to adapt under therapeutic pressure [42,43,44,45]. In contrast, eliminating cancer cells by triggering apoptosis will, in theory, reduce the chances of therapeutic resistance. The intrinsic apoptosis pathway is tightly regulated by a range of pro- and anti-apoptotic B-cell lymphoma 2 (BCL-2) family proteins [46]. Achieving apoptotic cancer cell death is reliant on breaching the apoptotic threshold, and upregulation of anti-apoptotic BCL-2 family proteins has been shown to promote tumor progression, androgen independence, and treatment resistance [47,48,49,50,51,52,53,54,55]. As such, targeting the intrinsic apoptosis pathway is an attractive therapeutic strategy for advanced prostate cancer, though molecular characterization and the identification of tumors with a dependency and/or vulnerability will be critical to best utilize this approach.

This review provides an up-to-date overview of cell death and apoptosis, specifically focusing on the intrinsic pathway. It summarizes the latest approaches for targeting the intrinsic apoptosis pathway with BCL-2 homology domain 3 (BH3) mimetics, and discusses how these strategies may be leveraged to treat prostate cancer.

## 2. Cell Death: Multiple Ways to Die

Cell death is a critical part of organismal homeostasis, maintaining balance between new cell generation by mitosis and the removal of damaged or unwanted cells. Historically, morphological classification has been used to characterize cell death into three main processes: apoptosis, necrosis, and autophagy (Table 1) [56]. However, in recent years, as understanding has developed, there has been a push to move away from morphological classification, and, since 2005, the Nomenclature Committee on Cell Death (NCCD) has proposed an updated classification of cell death modalities based on the mechanistic and requisite aspects of the process [57].

Cells can either die from accidental cell death (ACD) or regulated cell death (RCD) (Table 2). ACD is an uncontrolled process characterized by the sudden catastrophic demise of cells after exposure to a range of physical (e.g., extreme temperature), chemical (e.g., pH variation), or mechanical insults. In contrast, RCD is a highly coordinated process dependent on the activation of one or more genetically encoded signal transduction pathways. RCD can occur in two contrasting situations: (1) in response to intra- or extracellular perturbations when adaptive responses fail to restore cellular homeostasis, or (2) as part of routine physiological processes such as tissue development, turnover, and remodeling (often referred to as programmed cell death) [57,58]. There are a multitude of RCD mechanisms, with significant interconnectivity, all of which can exhibit a range of morphological features (from apoptotic to necrotic) (Table 2) [57].

Apoptosis, a form of RCD, is characterized by caspase-dependent proteolysis, endonucleolytic DNA fragmentation, membrane blebbing, and the formation of apoptotic bodies which are subsequently engulfed by resident phagocytes [59]. Apoptosis was historically thought to be an immunologically silent process, but it has become evident that this is not always the case [60]. Apoptosis can be triggered by two core pathways: extrinsic and intrinsic. The extrinsic pathway is stimulated by perturbations in the extracellular microenvironment, which trigger death receptors on the cell membrane (including Fas, TNF, and TRAIL), leading to activation of caspase 8 and subsequent activation of effector caspases, such as caspase 3. In ‘type I cells’, there is sufficient stimulation of the effector caspases to drive cell death, independent of the intrinsic pathway [61,62]. However, in ‘type II cells’, which includes most cancer cells, the extrinsic pathway relies on cleavage of BID by caspase 8 to form truncated BID (tBID). tBID subsequently translocates to the mitochondria and stimulates the intrinsic pathway by inducing BAX/BAK-dependent mitochondrial outer membrane permeabilization (MOMP), release of cytochrome c, and caspase 9-driven cell death [63,64,65].

Whilst deregulation of apoptosis is widely recognized to be a crucial part of tumorigenesis and treatment resistance, other forms of cell death may also play a role, and could be leveraged for therapeutic benefit. For example, necroptosis is a caspase-independent form of RCD, presenting with a necrotic morphotype, and may trigger anti-tumor immunity. Mechanistically, necroptosis critically depends on the activation of RIPK3 and MLKL [57]. Interestingly, RIPK3 has been shown to be downregulated in prostate cancer, and its overexpression suppressed prostate cancer cell migration and invasion [66]. Ferroptosis, another form of RCD, is characterized by iron-dependent lipid peroxidation, and a recent study demonstrated anti-tumor activity in prostate cancer cell line mouse xenograft models with ferroptosis inducing agents [67].

## 3. The Intrinsic Apoptosis Pathway

**Figure 1:**
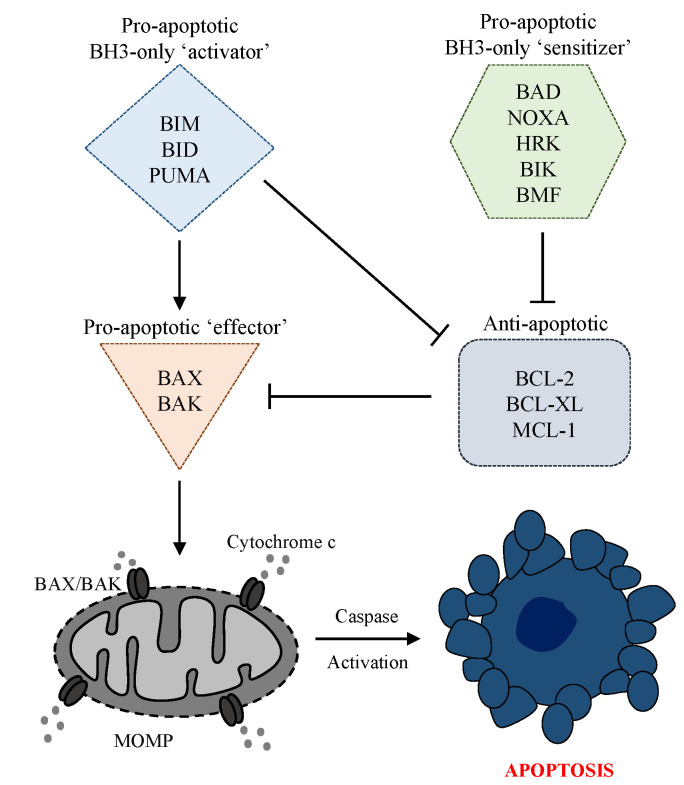
The intrinsic apoptosis pathway is tightly regulated by pro- and anti-apoptotic BCL-2 proteins which interact on the mitochondrial outer membrane. Pro-apoptotic BCL-2 proteins can be subcategorized as BH3-only ‘activators’ (BIM, BID, PUMA), BH3-only ‘sensitizers’ (BAD, NOXA, HRK, BIK, BMF) and pore-forming ‘effectors’ (BAK, BAX). Counteracting these are anti-apoptotic/pro-survival BCL-2 proteins including MCL-1, BCL-2, BCL-XL which sequester pro-apoptotic BCL-2 family members. BH3-only ‘activators’ promote apoptosis by directly engaging and activating BAX/BAK, as well as sequestering anti-apoptotic BCL-2 proteins. BH3-only ‘sensitizers’ are unable to directly engage BAX/BAX and primarily function by sequestering anti-apoptotic members. The induction of BH3-only proteins in response to cell death stimuli can trigger BAX/BAK homo-oligomerization, the formation of macropores, mitochondrial outer membrane permeabilization (MOMP), the release of cytochrome c and caspase driven apoptotic cell death.

The intrinsic apoptosis pathway, also known as the ‘stress’ or ‘mitochondrial’ apoptosis pathway, is tightly regulated by BCL-2 proteins; a family of pro- and anti-apoptotic proteins which share between one and four BCL-2 homology (BH) domains (BH1, BH2, BH3, BH4) and interact on the mitochondrial outer membrane [68]. The pro-apoptotic BCL-2 proteins can be subcategorized as BH3-only ‘activators’ (BIM, BID, PUMA), BH3-only ‘sensitizers’ (BAD, NOXA, HRK, BIK, BMF), and pore-forming ‘effectors’ (BAX, BAK). Counteracting these are the anti-apoptotic/pro-survival BCL-2 proteins, including MCL-1, BCL-2, BCL-XL, BCL-W, and BFL-1 (Figure 1) [69]. The balance between pro- and anti-apoptotic BCL-2 family proteins is critical in determining cell fate decisions, and manipulating this balance to breach the apoptotic threshold is an attractive strategy in cancer therapeutics [70].

A range of cellular stress and cell death stimuli, such as DNA damage (including that from DNA-targeting anti-cancer agents and radiation), replication stress, growth factor deprivation, and endoplasmic reticulum stress can initiate intrinsic apoptosis by inducing BH3-only ‘activator’ and/or ‘sensitizer’ proteins via transcriptional upregulation or post-translational modification [57,69]. For example, p53 induces PUMA and NOXA mRNA transcription in response to DNA damage, thereby priming the cell for apoptosis [71,72]. In response to these cell death stimuli, BH3-only ‘activator’ proteins (BIM, BID, PUMA) can bind directly to, and activate, the ‘effector’ proteins (BAX and/or BAK). In addition to direct stimulation of BAX and BAK, the BH3-only ‘activator’ proteins also bind to and sequester anti-apoptotic/pro-survival BCL-2 family proteins.

BH3-only proteins that lack the capability to directly engage BAX and BAK are termed ‘sensitizer’ proteins (BAD, NOXA, HRK, BIK, BMF), and predominantly act by inhibiting anti-apoptotic proteins [73]. Anti-apoptotic members of the BCL-2 family (MCL-1, BCL-2, BCL-XL, BCL-W, BFL-1) contain all four BH domains and enact their pro-survival function by directly binding to, and sequestering, pro-apoptotic BCL-2 proteins (Figure 1). These pro-survival proteins contain a hydrophobic groove, formed by the first three BH domains (BH1-3), which is able to bind to the hydrophobic face of pro-apoptotic proteins in a highly selective manner. Of note, BH3-only proteins have varying binding affinities for different anti-apoptotic proteins, as they also do with pro-apoptotic ‘effector’ proteins [74].

Mounting evidence suggests that BH3-only proteins can also induce apoptosis by sequestering anti-apoptotic proteins away from BAK and BAX, allowing them to autoactivate, without the need for direct activation [75,76,77,78]. Once activated, BAX/BAK undergo homo-oligomerization and form macropores, leading to MOMP and subsequent release of cytochrome c from the mitochondrial intermembrane space. Cytochrome c binds to APAF-1 to form the apoptosome, which activates caspase 9. Caspase 9 subsequently activates the effector caspases, including caspase 3 and 7, which disassemble the cell in preparation for phagocytosis. Counteracting the apoptosome and caspase activation are a family of inhibitor of apoptosis (IAP) proteins, which can in turn be inhibited by Smac and Omi proteins [79].

Although effector caspases are widely acknowledged to precipitate apoptosis, their stimulation is not essential for the process, and inhibiting caspase activation does not prevent cell death [59,80]. Preventing post-mitochondrial caspase activation, either with genetic manipulation or pharmacological inhibitors, does, however, delay intrinsic apoptosis and promote conversion to other forms of RCD [59]. MOMP has historically been seen as the point of no return, but if permeabilization only occurs in a limited number of mitochondria, the activation of caspases is not sufficient to drive cell death; however, it has been shown to induce DNA damage and genomic instability [81].

The susceptibility of healthy cells to apoptosis varies greatly in normal tissues, depending on organ type and stage of development, and primarily relies on the balance between pro- and anti-apoptotic BCL-2 family proteins. In early life, during tissue development, MYC-driven transcription of pro-apoptotic genes primes cells for apoptosis, allowing the removal of unwanted or damaged cells generated by high proliferation [82]. This renders developing organs highly susceptible to various insults, and accounts for the high levels of neuro- and cardiotoxicity observed when young children are treated with DNA-damaging agents, including chemotherapy and radiation. Over time, during post-natal tissue development, post-mitotic tissues, including the heart and brain, become refractory to apoptosis, primarily due to downregulation of BAX and BAK [82,83]. At the other end of the spectrum, cells of hematopoietic lineage, including peripheral mononuclear, thymus, spleen, and bone marrow remain highly primed for apoptosis throughout life, which explains the high incidence of myelosuppression during cytotoxic chemotherapy [82]. The susceptibility of normal tissues to apoptosis must be considered when developing strategies to trigger this pathway in cancer therapy.

## 4. Evasion of Apoptosis in Cancer

Owing to oncogenic DNA damage, aberrant growth signals, and increased cell cycling, the transformation of a cell to a malignant state is inherently stressful, and primes cells for apoptosis, serving as a natural barrier to oncogenesis [84]. Many of the genes responsible for cell growth and proliferation are controlled by MYC, explaining the deregulation of this oncogene in the majority of malignancies, including prostate cancer [85]. *MYC* is one of the most highly expressed genes in prostate cancer, and has been shown to positively regulate the *AR* and its splice variants [86]. As previously discussed, *MYC* drives upregulation of pro-apoptotic proteins and contributes to apoptotic priming during neoplastic transformation. However, as tumorigenesis progresses, cancer cells acquire the means to circumvent apoptosis; evasion of apoptosis is an established ‘hallmark of cancer’ [87]. Cancer cells encounter cumulative stresses during tumor progression, including genomic instability, nutrient deficiency, and hypoxia, and to metastasize they must be refractory to detachment-induced apoptosis (anoikis) [88,89]. Cancer cells can adapt to escape these cell death stimuli by modifying their intrinsic apoptosis machinery, either by upregulation of anti-apoptotic, or downregulation of pro-apoptotic, BCL-2 proteins. A growing body of evidence suggests this is the case in prostate cancer, an aspect of which is discussed in detail later in this review. This can occur via a multitude of mechanisms, including gene amplification, chromosomal translocation, transcriptional/translational regulation, or post-translational modifications [90,91]. Post-translational modifications include phosphorylation and regulation of protein stability via the ubiquitin proteasome system, especially of MCL-1 and BIM, which are both characterized by rapid turnover and short half-life [91]. For example, phosphorylation of MCL-1 by GSK3 is known to promote its degradation by recruiting E3 ubiquitin ligases including beta-TRCP and FBW7 [92,93]. In contrast, a range of deubiquitinating enzymes, such as Usp9x, counteract this process and help stabilize MCL-1 [94].

## 5. Interrogating BCL-2 Protein Dependency

Given the abundance of BCL-2 proteins, along with their complex interactions and context-dependent activity, it is challenging to analyze mitochondrial apoptotic sensitivity with biochemical assays. Although the relative levels of BCL-2 proteins correlate with sensitivity to BH3 mimetics in some malignancies, this is not uniformly observed, and the measurement of RNA or protein levels does not account for protein activity or important interactions [95,96,97,98,99]. For example, in patients with chronic lymphocytic leukemia (CLL), low MCL1 expression and high BIM:MCL-1 or BIM:BCL-2 ratios correlate with increased response to navitoclax (BCL-2/BCL-XL/BCL-W inhibitor) [95]. On the other hand, in myeloma cells, the binding of BIM to BCL-XL and BCL-2, but not the expression pattern, is associated with sensitivity to ABT-737 (BCL-2/BCL-XL/BCL-W inhibitor) [97]. In view of this complexity, over the last 15 years, ‘dynamic BH3 profiling’ assays have been developed to functionally measure cellular response to defined pro-apoptotic signals, using a range of BH3 peptides, allowing investigators to: (1) assess cellular susceptibility to intrinsic apoptosis (‘priming’) and (2) evaluate dependencies on specific anti-apoptotic proteins [100,101]. As specific ‘sensitizer’ BH3-only proteins have different affinities for anti-apoptotic proteins, it is possible to use certain peptides, which mimic the activity of ‘sensitizer’ proteins, to elucidate the dependency of cells to specific anti-apoptotic proteins. For example, NOXA preferentially inhibits MCL-1, whereas HRK preferentially inhibits BCL-XL [100]. Other approaches to elucidate dependencies include utilizing CRISPR/cas9 genome-editing technology to silence specific anti-apoptotic BCL-2 proteins, or exposing tumor models to a range of BH3 mimetics (see below) [102,103]. Pre-treatment mitochondrial priming has been shown to correlate with response to cytotoxic chemotherapy in patients with ovarian cancer, multiple myeloma, and acute myelogenous and lymphoblastic leukemia [70]. Using these technologies to elucidate sensitivity to agents targeting the intrinsic apoptosis machinery may help identify predictive biomarkers for this strategy.

## 6. Targeting the Intrinsic Apoptosis Pathway with BH3 Mimetics

Recent advances in medicinal chemistry have enabled the development of agents targeting the intrinsic apoptosis pathway. IAP inhibitors and Smac mimetics are currently being evaluated in early phase trials, although the most clinically advanced agents are the BH3 mimetics, which this review will focus on. BH3 mimetics are a range of small molecules designed to mimic the binding of BH3-only proteins to the hydrophobic groove on anti-apoptotic BCL-2 proteins, thereby liberating BAX/BAK to spontaneously activate and/or release BH3-only proteins to exert their pro-apoptotic effect on BAX/BAK. Venetoclax, a BCL-2 inhibitor, is currently the only agent with FDA approval that directly targets the intrinsic apoptosis machinery. First generation ‘pan-BH3 mimetics’, such as AT-101 and obatoclax, inhibited all anti-apoptotic BCL-2 family proteins, though off-target dose-limiting toxicities halted their clinical development [104,105]. In fact, subsequent studies showed that AT-101 and obatoclax were not authentic BH3 mimetics, as their pro-apoptotic activity resulted from off-target effects [106]. Next generation BH3 mimetics, mirroring endogenous BH3-only proteins, have been developed to have varying affinities for specific anti-apoptotic proteins. ABT-737, the first authentic BH3 mimetic, was developed to selectively inhibit BCL-2, BCL-XL and BCL-W, and showed promising activity against lymphoma and small-cell lung carcinoma mouse xenograft models, providing proof of principle for this therapeutic approach [107]. However, owing to its unfavorable pharmacokinetic properties, an orally bioavailable successor, navitoclax (ABT-263), was developed [108]. Navitoclax entered clinical trials in 2005, and although its clinical development has been hindered by dose-dependent thrombocytopenia, related to the inhibition of BCL-XL on platelets, it continues to be evaluated for myelofibrosis, as well as a range of advanced solid tumors, primarily in combination with tyrosine kinase inhibitors [109,110].

### 6.1. Selective BCL-2 Targeting

To mitigate the toxicity associated with BCL-XL inhibition, venetoclax (ABT-199), a highly selective BCL-2 inhibitor, was developed [111]. Venetoclax demonstrated strong anti-cancer activity in a variety of hematological malignancies with manageable toxicity, and has subsequently been approved by the FDA as a single agent, or in combination with rituximab or obinutuzumab, for the treatment of CLL and small lymphocytic lymphoma, as well as in combination with azacitidine, decitabine, or low-dose cytarabine for the treatment of acute myeloid leukemia (AML) [111,112,113,114,115,116]. In relapsed CLL, using pooled data from 4 early-phase trials, single agent venetoclax induced objective responses in 75% of patients, including 22% who achieved a complete response [117]. For patients with untreated AML who were not eligible for standard induction therapy (due to comorbidities or >75 years of age), the combination of venetoclax and azacitidine improved median overall survival from 9.6 to 14.7 months compared to azacitidine alone, with an improvement in complete response rate from 17.9% to 36.7% [113]. The success of BCL-2 inhibition in hematological cancers has been attributed to the upregulation of, and dependency on, BCL-2. Multiple studies have shown high BCL-2 tumor cell expression associated with venetoclax sensitivity; however, this is not uniformly observed, suggesting this is not the only factor determining response [111,118,119,120,121,122]. For example, there is high and uniform expression of BCL-2 in follicular lymphoma, though response to venetoclax is limited, demonstrating that its predictive use is context dependent [118].

Although venetoclax has proven efficacious for hematological malignancies, relapse is common and multiple resistance mechanisms have been described, including mutations in *BCL-2*, upregulation of MCL-1 and BCL-XL, TP53 dysfunction, and *BAX* loss [123,124,125,126]. Moreover, BCL-2 inhibition appears to have limited efficacy in solid tumors. A high-throughput venetoclax drug screen in cell lines from 26 different solid cancer types revealed limited activity, apart from in a subset of small-cell lung cancer (SCLC), bone and nervous system tumors [127]. In SCLC, high BCL-2 expression was found to correlate with sensitivity to venetoclax in cell lines and patient-derived xenograft models [127]. Although single agent activity appears to be limited in solid tumors, several pre-clinical studies have shown efficacy for venetoclax in combination with other agents, and multiple early phase clinical trials are ongoing, including one for men with prostate cancer [128,129,130]. A phase I study, the first to evaluate venetoclax for solid tumors, showed promising clinical activity in combination with tamoxifen for estrogen receptor (ER) and BCL-2-positive breast cancer, although a subsequent phase II study showed no benefit from the addition of venetoclax to fulvestrant for patients with ER positive/HER2 negative breast cancer who had progressed on a CDK4/6 inhibitor [131,132].

### 6.2. Strategies for Selective BCL-XL and Dual BCL-XL/BCL-2 Targeting

In contrast to hematological malignancies, solid tumors, including prostate cancer, are more dependent on BCL-XL and MCL-1 for cell survival [133,134,135,136,137]. BCL-XL is highly expressed in a variety of solid malignancies, and its overexpression is associated with tumor progression and treatment resistance, making it a promising therapeutic target [138,139]. Pre-clinical studies have revealed anti-tumor activity in response to BCL-XL inhibition across a range of solid tumor cell lines and xenograft models, predominantly in combination with other agents [135,140,141,142,143,144,145,146]. As such, efforts are ongoing to develop strategies to target BCL-XL, which can circumvent on-target thrombocytopenia. ABBV-155 is an antibody drug conjugate linking a BCL-XL inhibitor with an anti-B7H3 antibody, thereby specifically targeting B7H3-expressing cancer cells and minimizing platelet destruction. A first-in-human study has shown anti-cancer activity and tolerable toxicity for advanced solid tumors, in combination with paclitaxel, and the expansion phase is ongoing [147]. Another approach to diminish on-target toxicity is to use proteolysis targeting chimera (PROTAC) technology that targets a protein for degradation, and therefore does not need to directly inhibit protein activity. PROTACs are bivalent small molecules composed of two ligands; one able to bind a target protein and the other able to recruit an E3-ligase, resulting in ubiquitination and degradation of the protein via the proteasome. DT2216 is a PROTAC that targets BCL-XL to the Von Hippel-Lindau (VHL) E3-ligase, and, because there is limited expression in platelets, it has been shown to reduce on-target thrombocytopenia in mouse models, with a first-in-human study ongoing [148,149]. Interestingly, DT2216 has been shown to induce apoptosis in tumor-infiltrating regulatory T-cells, and activate tumor infiltrating CD8+ cells, resulting in suppression of tumor growth in immunocompetent, but not-immunodeficient, mouse models, indicating that targeting BCL-XL may be a promising strategy in cancer immunotherapy [150].

AZD0466, a dual BCL-2/BCL-XL inhibitor (AZD4320) conjugated to a PEGylated poly-lysine dendrimer, has been developed to optimize drug release rate and achieve maximal therapeutic index in terms of efficacy and tolerability. It is currently being evaluated in a phase I clinical trial for patients with advanced hematological or solid tumors [151,152]. Palcitoclax (APG-1252), a novel dual BCL-2/BCL-XL inhibitor, has also been designed to reduce platelet toxicity, and a ‘3+3’ design phase 1 escalation study revealed favorable toxicity and anti-cancer activity with 3 out of 36 evaluable patients achieving a partial response, including in one patient with neuroendocrine prostate cancer [153].

### 6.3. Efforts to Target MCL-1

Developing high-affinity inhibitors of MCL-1 has proven more challenging than for BCL-2 and BCL-XL, although several novel compounds have recently entered first-in-human studies. A wide range of malignancies rely on MCL-1 for survival, and *MCL-1* is one of the most frequently amplified genes in human cancer [137]. Knockdown of *MCL*-1, using short hairpin RNA, resulted in a more pronounced reduction in proliferation in *MCL-1* amplified cell lines compared to non-amplified controls, indicating the functional relevance of this aberration [137]. In a range of hematological and solid cancers, MCL-1 has been implicated in tumor formation, cell survival, and treatment resistance, including resistance to BCL-XL and BCL-2 targeting [137,154,155,156,157,158,159,160,161]. The majority of pre-clinical studies have been conducted in hematological cancers, where a number of MCL-1 inhibitors have shown promising activity [162,163,164,165,166]. For example, MCL-1 inhibition led to significant induction of apoptosis in primary human multiple myeloma samples and amplification of 1q21 (containing *MCL-1*), which occurs in around 40% of newly diagnosed disease, correlated with sensitivity [167]. With respect to solid tumors, MCL-1 inhibition was effective in a subset of SCLC cell lines with high MCL-1 and low BCL-XL expression, including in a patient-derived cell line xenograft model in vivo [168]. In non-small cell lung cancer, genomic gains in *MCL-1* occur with high frequency during tumor evolution, and tumor progression in transgenic mice was delayed with genetic or pharmacological inhibition of MCL-1 [169]. Pre-clinical anti-cancer activity has also been reported, primarily in combination with other therapeutics, in melanoma, breast, and colorectal cancer [161,170,171,172].

MCL-1 inhibitors under evaluation in early phase clinical trials include AMG-176, AMG-397, AZD-5991, S64315, ABBV-567, and PRT1419 [162,163,164,165,166]. Preliminary phase I data for AMG-176 suggested the drug may have acceptable toxicity, but its development, along with that of AMG-397, has been halted due to concerns regarding cardiac toxicity [173]. MCL-1 plays a critical role in normal physiology, including cardiomyocyte mitochondrial homeostasis, and MCL-1 knockout in mice induces fatal cardiomyopathy, highlighting the challenge of identifying a therapeutic window for this target [174,175]. Clinical evaluation of AZD-5991, S64315, and ABBV-567 is ongoing in hematological malignancies, and PRT1419 is also being evaluated in advanced solid malignancies. It remains to be seen whether novel drug delivery technologies, such as antibody drug conjugates and PROTACs, will be required to reduce dose limiting toxicities of MCL-1 inhibition, similar to how they are being evaluated for targeting BCL-XL.

## 7. Evasion of Apoptosis in Prostate Cancer and Resistance to Established Therapies

**Figure 2:**
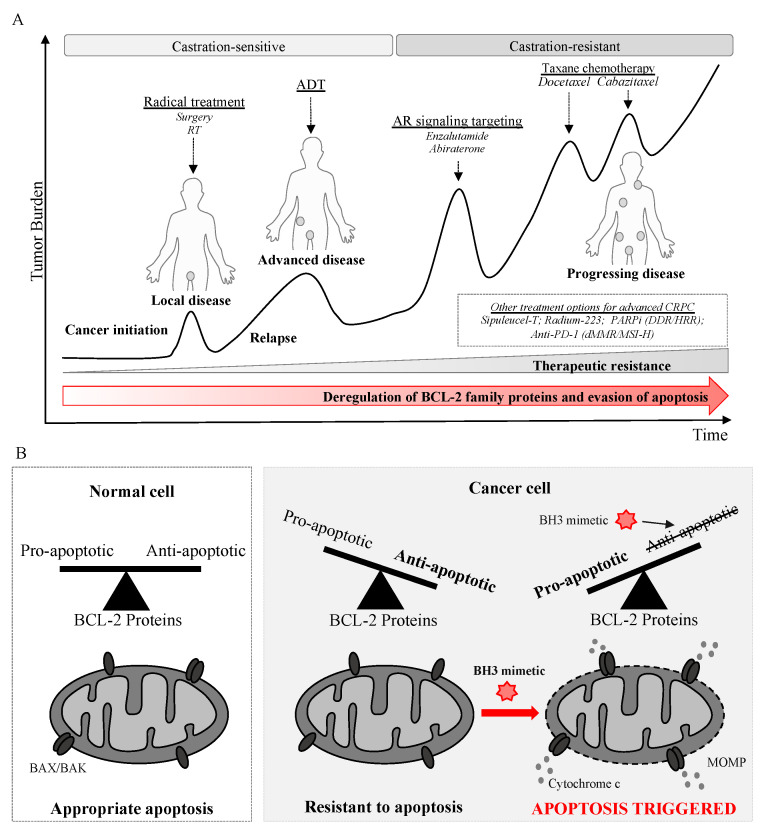
Evasion of apoptosis drives tumorigenesis, progression and therapeutic resistance in prostate cancer providing rationale for targeting anti-apoptotic BCL-2 proteins with BH3 mimetics. (**A**). An example of prostate cancer progression and treatment. Three-quarters of men are diagnosed with localized disease and can be treated with curative intent (prostatectomy or radiotherapy). However, an increasing percentage of men are being diagnosed with de novo metastatic disease and relapse occurs in 20 to 50% after radical treatment for early-stage disease. Most advanced prostate cancers respond to androgen deprivation therapy (ADT) but they inevitably progress from castration-sensitive to castration-resistant prostate cancer (CRPC). Approved therapies for advanced CRPC include androgen receptor (AR) signaling targeting, taxane-based chemotherapy and radium-223 but primary and acquired resistance is common. Tumors with aberrations in homologous recombination repair (HRR) and DNA damage response (DDR) genes can be treated with Poly(ADP-ribose) polymerase (PARP) inhibitors and anti-PD-1 therapy can be used for defective mismatch repair (dMMR) or microsatellite instability-high (MSI-H) disease. Treatment sequence can vary considerably between patients depending on the nature of disease; AR signaling targeting and docetaxel can be also used for castration-sensitive disease. (**B**). The balance of pro- and anti-apoptotic BCL-2 proteins is critical in determining cell fate decisions. BCL-2 proteins are finely balanced in normal cells which can initiate apoptosis appropriately on exposure to cell death stimuli. In cancer cells, BCL-2 proteins are deregulated, increasing the threshold for apoptosis and promoting cell survival. BH3 mimetics bind to anti-apoptotic BCL-2 proteins, lowering the threshold and can trigger BAX/BAK homo-oligomerization, mitochondrial outer membrane permeabilization (MOMP), the release of cytochrome c and induction of apoptotic cell death.

Deregulation and circumvention of apoptosis contributes to prostate cancer tumorigenesis, progression, and therapeutic resistance (Figure 2).

### 7.1. BCL-2 Proteins in Prostate Cancer

A number of immunohistological studies have shown high levels of MCL-1 and BCL-XL in prostate cancer, but BCL-2 appears to be expressed at lower levels, with more interstudy variability [47,176,177,178,179,180,181,182,183,184]. Published in 1996, Krajewska and colleagues were the first to undertake a comprehensive immunohistochemical analysis of the three major anti-apoptotic BCL-2 proteins in prostate cancer [177]. Immunohistochemistry for BCL-2, BCL-XL, and MCL-1 was undertaken for 64 cases of prostate adenocarcinoma, including eight bone and eight lymph node metastases. A total of 24 cases of prostatic intraepithelial neoplasia (PIN), largely accepted as a precursor lesion to invasive adenocarcinoma, were also analyzed. Lesions were defined as positive if at least 1% of tumor cells expressed the protein of interest. BCL-2 was positive in 25% of adenocarcinomas, with increasing frequency in high grade tumors and nodal metastases [177]. Conversely, a recent study using same patient matched samples showed no significant change in BCL-2 levels between CSPC and CRPC [185]. BCL-XL was found in all adenocarcinoma samples, with a higher immunointensity and percentage of positive cells in high grade primary tumors and metastases, compared to PIN and low grade tumors. MCL-1 was detected in 81% of adenocarcinomas, compared to 38% of PIN cases, with an increasing percentage of positive cells in high grade tumors and metastases. The pro-apoptotic pore forming ‘effector’ BAX was detected in all 88 samples, 86 of which contained >25% of positive staining tumor cells, suggesting that the majority of prostate cancers may still have the core machinery to undergo apoptosis if the apoptotic threshold is breached [177].

In another study, a post-mortem analysis of 185 metastatic lesions from 44 patients with heavily pre-treated metastatic prostate cancer revealed high levels of anti-apoptotic proteins, albeit with significant intra-patient heterogeneity between bone and soft tissue metastases [186]. In addition, pro-apoptotic BH3-only proteins have been implicated in prostate cancer, a topic which has been reviewed extensively elsewhere [187]. In summary, immunohistochemical evidence suggests that BCL-2 proteins play an important role in prostate cancer progression as the tumor becomes more resistant to cell death stimuli. Further studies with matched same patient diagnostic and metastatic sample analysis are required to confirm these findings and elucidate when these changes occur during tumor evolution. This will be important in determining the clinical translatability of targeting these proteins in advanced prostate cancer, although, as discussed, changes in BCL-2 proteins are dynamic, and this cannot be evaluated with immunohistochemical studies.

### 7.2. Resistance to Established Therapies

Established therapies for prostate cancer enact their therapeutic activity, to some extent, by the induction of apoptosis, and several studies have suggested that BCL-2 proteins play an important role in therapeutic resistance.

#### 7.2.1. Resistance to ADT

The majority of advanced prostate cancers initially respond to ADT, though they inevitably develop into CRPC, with poor prognosis. As such, the development of strategies to prevent and treat lethal CRPC remains of high clinical importance. A number of immunohistochemical and functional studies have associated anti-apoptotic proteins with castration resistance, with most data focusing on BCL-2 [47,49,178,188,189,190,191]. In the hormone sensitive LNCaP prostate cancer cell line model, upregulation of BCL-2 is required for progression from an androgen-dependent to androgen-independent state, and overexpression of BCL-2 confers resistance to androgen depletion, protecting cells against apoptosis [49,51,192]. Similarly, upregulation of BCL-2 mRNA and protein expression was observed in LNCaP, LAPC4, and LAPC9 CRPC xenograft models with acquired resistance to ADT, aligning with previous data that androgens suppress BCL-2 expression [191,193]. Importantly, the same was not observed in a VCaP xenograft model, highlighting the inherent diversity of resistance mechanisms, the challenges of inter- and intra-patient heterogeneity, and the importance of patient selection for therapeutics [191]. MCL-1 has also been implicated in resistance to ADT, and is upregulated in androgen deprived prostate cancer cells, preventing the induction of apoptosis [188]. Furthermore, high BCL-XL expression has been associated with poor prognosis and the development of CRPC [47]. The pro-apoptotic pore forming ‘effectors’, BAX and BAK, are expressed at higher levels in localized prostate cancer compared to CRPC, suggesting their downregulation may be associated with resistance to ADT; however, the study did not include matched same patient CSPC and CRPC samples [178].

#### 7.2.2. Resistance to AR Signaling Inhibition

Although the introduction of multiple agents that potently target the AR signaling axis (including abiraterone, enzalutamide and apalutamide) has improved outcomes for men with advanced prostate cancer, primary and acquired resistance remains common. Potent AR signaling inhibition has, in part, been shown to work via the induction of apoptosis, and BCL-2 is both induced by enzalutamide treatment and upregulated in castration resistant models with primary or acquired resistance to enzalutamide [191,194,195,196]. However, an analysis of same patient diagnostic and CRPC samples, from 20 patients with resistance to AR signaling axis targeting (7 of which had enzalutamide), showed no significant difference in BCL-2 protein levels [185].

Established resistance mechanisms that induce AR reactivation include *AR* amplification, *AR* mutation, increased AR ligand availability, and the generation of constitutively active AR splice variants; however, a subset of tumors are able to progress without relying on ARs to proliferate, termed AR pathway-independent prostate cancer [12]. Typically, this has been associated with small cell/neuroendocrine trans-differentiation with loss of AR expression, and evidence suggests that BCL-2 is highly upregulated in small cell neuroendocrine prostate cancer, compared to AR positive disease [197]. Furthermore, mounting data suggests that resistance to AR targeting can occur via an AR ‘indifferent’ state, where AR remains expressed but is not relied upon for cell proliferation [198,199,200,201]. Interestingly, two cell line models developed with acquired resistance to AR targeting, with AR indifference, were shown to have upregulation of MCL-1 and increased resistance to taxane chemotherapy [48].

#### 7.2.3. Resistance to Chemotherapy

Targeting microtubules with taxane-based chemotherapy has been a mainstay of treatment for advanced prostate cancer since the approval of docetaxel for advanced CRPC in 2004 [15]. Subsequent studies have shown increased benefit when used earlier in the disease course for advanced CSPC [202]. Ten years ago, cabazitaxel was introduced as a second-line taxane chemotherapy for patients who had progressed on or after docetaxel [17]. Although taxane-based chemotherapy has improved outcomes for men with this lethal disease, primary and acquired resistance is common, and the survival advantage is modest [15,17,27]. As with hormonal treatment, taxane chemotherapy is postulated to, at least to some extent, exert its therapeutic benefit by inducing apoptosis, and elevated serum caspase-cleaved cytokeratin levels are observed in men treated with docetaxel for CRPC [40,203,204,205]. Microtubule targeting agents, including taxanes, prolong mitosis and promote apoptosis via degradation of MCL-1 [206]. MCL-1 has also been shown to protect prostate cancer cells from chemotherapy-induced DNA damage and cell death [53]. In addition, downregulation of anti-apoptotic BCL-XL in LNCaP and PC-3 prostate cancer cells increases sensitivity to multiple chemotherapeutic agents, whereas overexpression of BCL-XL is cytoprotective [52]. Conversely, high BCL-2 expression has been associated with improved outcome in men treated with taxane chemotherapy for CRPC, and is downregulated in response to docetaxel [178,207]. Taxanes are known to induce phosphorylation of BCL-2, reducing its anti-apoptotic function, which may explain this observation [178,208]. BCL-2 proteins have also been implicated in resistance to radiotherapy in prostate cancer, and an elevated BCL-2/BAX ratio is associated with radioresistance [209,210].

In conclusion, deregulation of the intrinsic apoptosis machinery contributes to the development of CRPC and resistance to established therapies, suggesting that the utilization of therapies to target this pathway and breach the apoptotic threshold is an attractive strategy to treat this lethal disease.

## 8. Targeting the Intrinsic Apoptosis Pathway in Prostate Cancer with BH3 Mimetics

Inhibiting the three major anti-apoptotic proteins (BCL-2, BCL-XL, and MCL-1) is highly effective in driving apoptotic cell death; however, this is unlikely to be feasible in the clinic as significant toxicities are expected [211,212,213]. In fact, prior to the development of BH3 mimetics with high specificity, ‘pan-BH3 mimetics’ with broad engagement of multiple anti-apoptotic BCL-2 proteins were evaluated in pre-clinical studies, with promising results, including in prostate cancer [188,214,215]. However, these agents have lower affinity and specificity for their targets. The pan-BCL-2 inhibitor AT-101 was evaluated in early phase clinical trials for prostate cancer, but off-target gastrointestinal toxicities were dose limiting, and there was no significant anti-cancer activity [104,216,217]. As discussed, in view of this, these agents are no longer being evaluated in clinical trials, and have been superseded by highly selective BH3 mimetics with higher affinity and fewer off target effects.

Although a proportion of prostate cancers with significant addiction to a specific anti-apoptotic protein may respond to a single agent BH3 mimetic, it is likely that combination therapies will be needed to lower the apoptotic threshold and increase the response rate. As with other solid malignancies, BCL-XL and MCL-1 appear to be more important than BCL-2 for cell survival in prostate cancer, although it is likely there is significant inter- and intra-patient heterogeneity [186,211]. As discussed, engagement of intrinsic apoptosis machinery is implicated in the efficacy of, and resistance to, many established therapies in prostate cancer, including ADT, AR signaling axis targeting, and chemotherapy (Figure 2). In this space, several pre-clinical studies have shown additional efficacy when these treatments are combined with BH3 mimetics.

Using LNCaP, C4-2, and 22Rv1 prostate cancer cells, synergism has been shown between enzalutamide and a range of BH3 mimetics with the induction of apoptosis [196]. Predictably, there were discrepancies in the efficacy of different combinations between the cell lines, signifying the importance of identifying predictive biomarkers to enrich response. Synergism between enzalutamide and navitoclax (BCL-2/BCL-XL/BCL-W inhibitor) was observed in all three cell lines, with the most marked effect in LNCaP cells, which also showed moderate sensitization with the addition of venetoclax (BCL-2 inhibitor). In contrast, 22Rv1 cells were most sensitive to the addition of A-1210477, an MCL-1 inhibitor [196]. In another study, the combination of enzalutamide and navitoclax (ABT-263) increased sensitivity in both enzalutamide-sensitive and (primary and acquired) -resistant prostate cancer cells, with elevated proteasomal degradation of AR and AR splice variant 7 (AR-V7) [218]. In vivo activity was also observed when 22Rv1 cell line mouse xenografts, which have primary resistance to enzalutamide, were treated with the combination of enzalutamide and navitoclax [218].

In keeping with elevated BCL-2 dependence with AR inhibition, in another study, venetoclax (BCL-2 inhibitor) was one of two agents used to specifically target enzalutamide resistant cells in a large-scale pharmacology screen [185]. The authors, and others, have validated these findings and shown enhanced anti-tumor activity and delayed resistance with the addition of venetoclax to enzalutamide in LNCaP derived CRPC AR-positive mouse xenograft models, suggesting this combination could be used to prevent and treat enzalutamide resistant CRPC [191]. In light of these findings, this combination is currently being evaluated in a phase Ib/II clinical trial for men with metastatic CRPC (NCT03751436). In contrast, when evaluating the AR negative LAPC9 CRPC model which has primary resistance to enzalutamide, in vivo anti-tumor activity was seen with single agent venetoclax, but there was no additional benefit with the addition of enzalutamide in organoid studies [191]. To our knowledge, NCT03751436 is the only clinical trial currently evaluating a selective BH3 mimetic specifically for prostate cancer. A phase Ib/II clinical trial assessing the combination of navitoclax and abiraterone, with or without hydroxychloroquine, was initiated in 2013 for men with metastatic CRPC, but was later terminated, as the investigator left the organization with insufficient data to assess outcome (ClinicalTrials.gov, NCT01828476).

De novo small-cell neuroendocrine prostate cancer (SCNPC) is extremely rare, accounting for <1% of prostate cancer at diagnosis [219]. However, as discussed, under potent AR signaling blockade, lineage plasticity and trans-differentiation from AR positive adenocarcinoma to an AR negative small cell/neuroendocrine phenotype occur in a subset of tumors. These tumors respond poorly to conventional therapies and are associated with poor prognosis. In keeping with upregulation of BCL-2 in small cell tumors, venetoclax (BCL-2 inhibitor) was shown to be more effective in SCNPC cells compared to AR-positive lines [197]. The authors subsequently treated five SCNPC patient-derived xenograft models with high expression of BCL-2 with navitoclax (BCL-2/BCL-XL/BCL-W inhibitor), observing two responses.

With respect to synergy with chemotherapy, navitoclax (ABT-263) sensitized PC3 prostate cancer cells to docetaxel in vitro and in vivo, with the predominant impact through inhibition of BCL-XL [220]. Similarly, navitoclax and ABT-737, the predecessor of navitoclax, have been shown to increase apoptotic cell death in LNCaP prostate cancer cells in combination with taxane-based chemotherapy (docetaxel and paclitaxel) [221,222]. Additionally, direct and indirect knockdown of MCL-1 has been shown to sensitize prostate cancer cells to anti-mitotic agents, including docetaxel, suggesting that MCL-1 targeting may also synergize with chemotherapy [53,223,224]. Interestingly, BCL-2 inhibition, with venetoclax or siRNA, sensitizes taxane-resistant prostate cancer cells (PC3 and DU145) to cisplatin chemotherapy, with BCL-2 overexpression reversing this effect [207].

Along with the established therapies, a number of cell survival signaling pathways are known to influence the intrinsic apoptosis pathway in cancer, some of which are emerging as important targets in prostate cancer. For example, PI3K/AKT pathway activation contributes to proliferation, progression, and treatment resistance in prostate cancer. Genomic aberrations in the PI3K/AKT signaling pathway are common in primary prostate cancer, and enriched in advanced CRPC (approximately 20 and 50% respectively) [225,226,227]. Pathway activation is most often seen as a result of PTEN loss, occurring in around 40% of advanced CRPC. PTEN negatively regulates PI3K/AKT signaling, and its loss is emerging as a predictive biomarker for AKT inhibition. Recently published, the IPATential150 phase III trial showed a significant improvement in radiographic progression-free survival with the combination of the AKT inhibitor ipatasertib and abiraterone, versus abiraterone alone, in men with PTEN loss advanced CRPC [34]. Importantly, AKT is able to promote cell survival by phosphorylating BAD and inhibiting its pro-apoptotic function, thereby increasing the apoptotic threshold [196,228,229]. In keeping with this, the combination of a BCL-XL inhibitor (A-1331852) with an AKT (ipatasertib) or pan-PI3K inhibitor (buparlisib) was synergistic in PTEN deficient PC3 and LNCaP prostate cancer cell lines [230]. PTEN proficient DU145 cells were resistant to the combination; however, it should be noted that DU145 cells have reduced sensitivity to BH3 mimetics, which may be accounted for by the absence of BAX expression [220,221].

In addition, the Ras/Raf/MEK/ERK pathway promotes cell survival and has been shown to inhibit apoptosis by multiple mechanisms, including phosphorylation of BAD, suppression of BIM expression, and stabilization of MCL-1 [231,232,233]. Aberrations in this pathway are infrequently found in prostate cancer, but do correlate with progression and treatment resistance, providing pre-clinical rationale for targeting this pathway in combination with BH3 mimetics in this subset of tumors [234,235]. Furthermore, several receptor tyrosine kinase inhibitors, including erlotinib, lapatinib, cabozantinib, and sorafenib have been shown to increase MCL-1 degradation, resulting in robust apoptotic cell death when combined with ABT-737 in prostate cancer cells, both in vitro and in vivo. Interestingly, this was independent of the PI3K/AKT and Ras/Raf/MEK/ERK pathways, and instead driven by the integrated stress response and NOXA-dependent MCL-1 degradation, which was mediated by the E3 ubiquitin ligase MARCH5 [211,236]. These therapies may target MCL-1 more selectively in tumor cells, and could help circumvent the toxicity expected with direct MCL-1 inhibition in combination with BCL-XL/BCL-2 targeting. Aside from BH3 mimetics, there are limited data for other agents directly targeting the intrinsic apoptosis pathway in prostate cancer, and further studies are warranted. IAP family protein levels, including survivin, are known to be elevated in prostate cancer, and targeting survivin resulted in tumor regression in PC-3 cell line xenograft mouse models [237,238].

## 9. Future Perspectives and Conclusions

The eradication of prostate cancer cells by triggering the intrinsic apoptosis pathway is a promising new therapeutic strategy for prostate cancer. Driving apoptotic cell death, as opposed to growth inhibition, should reduce the chances of cancer cell adaptation and therapeutic resistance. Many of the established therapies for prostate cancer are known to lower the apoptotic threshold, providing strong biological rationale for combining these agents with BH3 mimetics. In addition, other cell survival pathways, such as PI3K/AKT signaling, are becoming important targets in prostate cancer, and drive cell survival, to some extent, by modulating intrinsic apoptotic machinery. BH3 mimetics have shown strong anti-cancer activity in hematological malignancies, and venetoclax, a BCL-2 inhibitor, is now approved for CLL and AML. Solid tumors, including prostate cancer, appear to be more reliant on BCL-XL and MCL-1 for cell survival. Efforts to target these proteins are ongoing, although the development of drug-related adverse events, including dose-limiting thrombocytopenia and cardiac toxicity, has slowed their clinical development. Novel drug delivery technologies, such as antibody drug conjugates (e.g., targeting PSMA or B7H3) or PROTACs, may help mitigate toxicities and induce cancer-specific cell death. Early-phase clinical trials evaluating BH3 mimetics are ongoing, though only one study is specifically focused on prostate cancer. Although there is good pre-clinical rationale for the use of these agents in prostate cancer, well designed biomarker-driven clinical trials are required to evaluate whether these strategies will improve outcomes for men with this lethal disease. In this space, the use of molecular characterization and the identification of tumors with a dependency on the intrinsic apoptosis pathway will be critical to the successful clinical implementation of these agents.

## Figures and Tables

**Table 1:** Historical classification of cell death based on stereotypical morphology.

Morphotype	Stereotypical Morphological Changes	Diagram
Apoptosis (Type 1 cell death)	Cytoplasmic shrinkage. Chromatin condensation. Plasma membrane blebbing. Formation of apoptotic bodies. Phagocytic engulfment of apoptotic bodies. Degradation of apoptotic bodies in lysosomes.	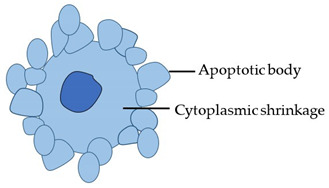
Autophagy (Type 2 cell death)	Extensive cytoplasmic vacuolization. Formation of autophagosomes. Fusion of autophagosomes with lysosomes.	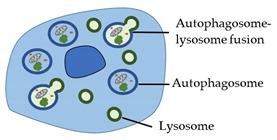
Necrosis (Type 3 cell death)	Cytoplasmic and organelle swelling. Rupture of plasma membrane. Release of cellular contents. Historically defined as cell death in the absence of apoptotic or autophagic morphological features.	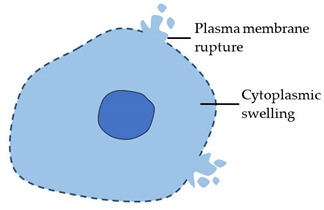

**Table 2:** Mechanistic classification of cell death (adapted from Galluzzi et al.; 2018) [57].

Cell Death Type	Definition
Accidental cell death (ACD)	Rapid and uncontrolled cell death triggered by extreme physical, chemical, or mechanical insults and characterized by plasma membrane rupture.
Regulated cell death (RCD)	Highly coordinated cell death, dependent on the activation of one or more signal transduction pathways. RCD can be subdivided into numerous subroutines with significant interconnectivity, all of which can present with a range of morphological features (apoptotic to necrotic) and immunomodulatory effects. Subroutines include intrinsic apoptosis, extrinsic apoptosis, mitochondrial permeability transition (MPT)-driven necrosis, necroptosis, ferroptosis, pyroptosis, parthanatos, entotic cell death, NETotic cell death, lysosome-dependent cell death, autophagy-dependent cell death, and immunogenic cell death (see NCCD classification, Galluzzi et al.; 2018).
Programmed Cell Death (PCD)	Regulated cell death that occurs as part of normal physiological processes.
